# Arterial Stiffness and Subclinical Inflammation in Children with Familial Mediterranean Fever: A Comprehensive Analysis

**DOI:** 10.3390/children12020232

**Published:** 2025-02-14

**Authors:** Nadide Melike Sav, Hasan Baki Altinsoy, Betul Turen, Ayşe Gökçe

**Affiliations:** 1 Department of Pediatric Nephrology, Duzce University, Duzce 81620, Turkey; 2 Department of Radiology, VM Medical Park Bursa, Bursa 16220, Turkey; hasanaltinsoy@duzce.edu.tr; 3 Department of Pediatrics, Dortcelik Pediatric Hospital, Bursa 16120, Turkey; betul.turen@saglik.gov.tr; 4 Department of Radiology, Bursa Yuksek Ihtisas Egitim ve Arastirma Hastanesi, Bursa 16310, Turkey; ayse.gokce2@saglik.gov.tr

**Keywords:** arterial stiffness, children, familial mediterranean fever, inflammation, intima–media thickness, real-time tissue elastography

## Abstract

**Background/Objectives**: Familial Mediterranean fever (FMF) is a chronic autoinflammatory disease. Throughout the disease, subclinical inflammation persists into the remission period. It is known that chronic inflammation causes endothelial dysfunction and, as a consequence, arterial stiffness occurs. In this study, carotid and aortic intima–media thicknesses (IMT) and arterial stiffness were measured in FMF patients to evaluate the risk of possible vascular damage due to chronic inflammation. **Methods**: The study included pediatric patients with FMF who had been in remission for a minimum of 3 months. Carotid and aortic IMT and arterial stiffness measurements were conducted using sonoelastography. The acute-phase reactants were also evaluated in all participants. **Results**: Carotid artery stiffness measurements by strain elastography were significantly higher in the patient group than in the control group. However, the aortic and carotid IMT were similar between the two groups. The acute-phase reactants were significantly higher in the patient group than in the control group. **Conclusions**: This study demonstrated that arterial stiffness increased in pediatric FMF patients. According to the results of the present study, the effects of chronic inflammation on arterial tissues may lead to atherosclerotic changes in the later stages of the disease and may pose a risk for coronary diseases. Arterial ultrasonographic and elastographic measurements to be performed periodically in children with FMF are noninvasive methods that can be used to evaluate the course of endothelial damage. We aimed to show that arterial stiffness may be a marker of early cardiovascular disease.

## 1. Introduction

Familial Mediterranean fever (FMF) is a genetic disease characterized by inflammatory attacks, primarily affecting the serous membranes of the peritoneum, pleura, and joints [1,2]. The gene responsible for FMF, identified by the International FMF Consortium, is designated as the MEFV gene. This gene encodes the pyrin/marenostrin protein and plays a pivotal role in regulating the inflammatory response [2,3]. Pyrin mutations trigger the release of interleukin (IL)-1β through caspase-1 activation, leading to inflammatory attacks [4].

FMF follows a pattern of periodic attacks; however, 30–90% of patients experience ongoing subclinical inflammation even in attack-free periods [5]. In a significant number of patients, inflammatory markers such as C-reactive protein (CRP) and serum amyloid A (SAA) persist at elevated levels even during periods of remission [6,7]. Furthermore, other proinflammatory cytokines, such as IL-6, IL-8, IL-12, and IL-18, are active during this period and contribute to the persistence of chronic inflammation [5]. This continuous inflammatory process can lead to long-term damage to various tissues, particularly vascular structures.

Arterial wall strain elastography is a recommended technique for investigating vascular disorders in chronic diseases involving multiple organs [8]. This technique qualitatively and quantitatively assesses tissue elasticity resulting from compression and stress of the target vessel using an ultrasound probe. The degree of arterial stiffness is determined by recording radiofrequency changes before and after compression. In this way, information can be obtained about the damage caused by the disease on the vessels [9].

Endothelial dysfunction and arterial stiffness are among the most significant vascular complications resulting from chronic inflammation in FMF patients [10]. Endothelial damage is considered one of the early indicators of atherosclerosis, and the deterioration of vascular structure triggered by subclinical inflammation can result in increased arterial stiffness [11]. Arterial stiffness is recognized as an important marker of early cardiovascular disease, and this risk is elevated in patients with chronic systemic inflammatory disorders such as FMF.

Studies conducted on FMF patients have shown an increase in the intima–media thickness (IMT) and related vascular stiffness from childhood onward [12]. Bilginer et al. reported that increased IMT in pediatric FMF patients was associated with persistent inflammation, even during remission periods [13]. These findings indicated that FMF patients are at increased cardiovascular risk owing to arterial stiffness and vascular damage caused by chronic inflammation.

The objective of this study was to evaluate the effect of subclinical inflammation on arterial structures during the remission period in pediatric FMF patients. For this purpose, the carotid and aortic IMT and carotid artery stiffness were measured in patients and controls using both B-mode and strain elastography. Furthermore, the duration of the disease, the number of attacks, and the levels of phase reactants such as CRP, erythrocyte sedimentation rate (ESR), fibrinogen, and SAA were also evaluated.

## 2. Materials and Methods

This study included 43 pediatric patients diagnosed with FMF who were younger than 18 years of age and 50 controls. The control group consisted of healthy volunteers who applied to the outpatient clinic, did not show any health problems, and were comparable to the patient cohort in terms of age and gender and whose laboratory test results were within normal limits.

Approval for the study was obtained from the Faculty of Medicine Clinical Research Ethics Committee (Date: 23 May 2022, Decision No: 2022/93). All participants and their families signed an informed consent form. The study was based on the ethical principles set forth in the Declaration of Helsinki. FMF was diagnosed according to the Tel-Hashomer diagnostic criteria [14]. All examinations and imaging were performed when the patients had been in remission for at least 3 months. Molecular genetic testing was performed on all patients diagnosed with FMF. The MEFV mutations were investigated using polymerase chain reaction (PCR) restriction fragment length polymorphism (PCR–RFLP) and the reverse hybridization assay (FMF StripAssay). Patients were regularly given appropriate doses of colchicine.

The exclusion criteria were as follows: aged older than 18 years, the presence of active infection, autoimmune and vasculitic diseases, congestive heart failure, diabetes, hypertension, and obesity (body mass index at or above the 95th percentile for sex and age). Children who were unable to comply with the measurements and those who did not provide informed consent were also excluded.

The blood and urine samples obtained from the patients were collected in accordance with the relevant protocols, and all tests were performed simultaneously. In addition to routine blood tests, the erythrocyte sedimentation rate (Westergren method) and the acute-phase reactants such as SAA (Immunoassay method), CRP (Turbidimetric immunoassay method), and fibrinogen (Coagulometric method) levels were also studied in all groups.

### 2.1. B-Mode Ultrasonographic and Elastography Evaluation

B-mode and elastographic assessments were performed by a radiologist with 6 years of sonoelastography experience using a digital sonography unit (LOGIQ S8; GE Healthcare, Wauwatosa, WI, USA) equipped with real-time tissue elastography software (Version R3). The B-mode and strain elastography (SE) images were obtained with a broad-spectrum linear transducer (9 L-D (2–8 MHz)). Since SWE was affected by vascular pulsation and could not be applied to a tiny area, such as the vessel wall, SE was applied to the carotid intima–media layer. The ultrasound gain, depth, focal points, and transducer frequency settings were kept constant in all the image scans. A color map representing tissue elasticity was superimposed on the grayscale sonographic image, with blue indicating the most elastic tissues, green-yellow indicating tissues with intermediate elasticity, and red indicating the least elastic tissues. The images obtained were stored in the archive.

**Figure 1:**
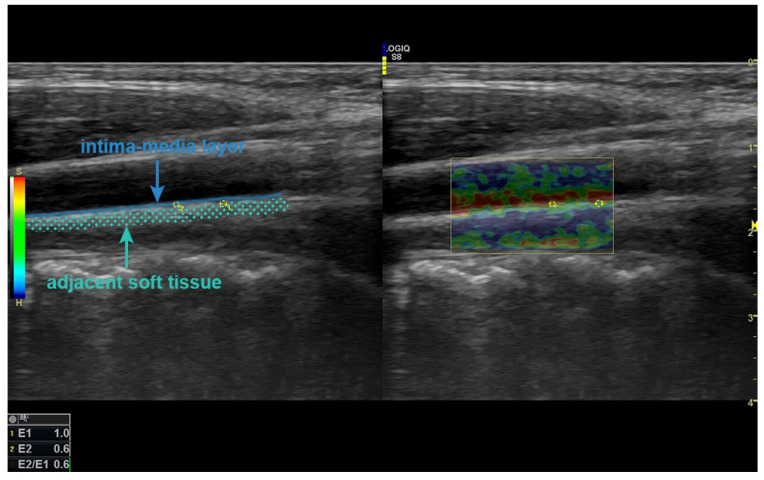
In SE examination, ROIs placed at the same depth in the intima–media layer and adjacent soft tissue on the posterior wall, 2 cm distal from the level of the left CCA bulb, are seen. SE: strain elastography, ROI: region of interest, CCA: common carotid artery.

The examinations were performed with the subjects in the supine position. IMT was measured on the posterior wall approximately 2 cm distal to the left carotid bulb in the longitudinal plane on the grayscale. SE was applied to the posterior wall 2 cm distal to the carotid bulb, including the intima–media layer and adjacent soft tissue. The region of interest (ROI) for measurement was sized not to exceed the carotid intima–media complex and was placed as centrally as possible. After this ROI area was set to the intima–media layer, another ROI area of similar diameter was placed in the surrounding tissue at the same depth (Figure 1). During the examination, two radiologists blinded to the patient and control groups decided the final result by consensus decision. The strain index (SI) was automatically calculated by the device software from the ratio of these two ROIs. Compared with the common carotid artery, the abdominal aorta was more difficult to assess because of its deep location and the superposition of gas distension, especially in the intestines. In addition, since the manual compression required for SE could not be achieved, sonoelastographic evaluation was not performed, and only the IMT was measured from the posterior wall at the infrarenal level.

### 2.2. Statistical Analysis

The distributions of the quantitative variables were examined using Shapiro–Wilk tests, normality plots, and skewness/kurtosis statistics. The mean standard deviation (SD) was calculated for the quantitative variables that fit a normal distribution. The median (interquartile range (IQR)) was given for nonnormally distributed variables. Sex and the presence of genetic mutations were reported by frequency (%).

The demographic characteristics and elastography findings were compared between the patient and control groups using the Student’s *t*-test, the Mann–Whitney U test, and the Pearson chi-square test concerning the distribution and type of variable of interest. The correlation coefficients were interpreted as suggested by Mukaka et al. [15]. The relationships between categorical variables were examined with Pearson Chi-Square or Fisher’s Exact tests, depending on the expected value rule. A *p*-value < 0.05 was considered statistically significant. All the statistical analyses were performed using IBM SPSS Statistics 22.0 (IBM Corp. Released 2013. IBM SPSS Statistics for Windows, Version 22.0. Armonk, NY, USA: IBM Corp.).

## 3. Results

The median age was 9.1 years (IQR: 6; 14) in the patient group and 10.8 years (IQR: 7; 13) in the control group. In the patient group, 48.8% were boys (*n* = 21) and 51.2% were girls (*n* = 22). However, in the control group, 42% were boys (*n* = 21), and 58% were girls (*n* = 29). There was no significant difference between the patient and control groups in terms of gender. Patient and control groups were similar in terms of age, sex, weight, and height. (Table 1). The median disease duration was 3.1 years (IQR: 2; 6, range: 1–10) in the patient group. The median number of attacks experienced by patients following a diagnosis of FMF was six. The clinical characteristics of the patients are given in Table 2.

Three patients were heterozygous and one patient was homozygous for any of the A744S, E167D, F479L, or A89T mutations; two patients were heterozygous for L110P; three patients were heterozygous for V726A; four patients were heterozygous for P369S, R408Q or E148Q; one patient was heterozygous for M680I; 11 patients were heterozygous and homozygous for M694V; and 21 patients were heterozygous and 12 homozygous genotypes were detected for R202Q. A total of 18 patients (41.9%) were compound heterozygous, 13 patients (30.2%) were heterozygous, and 12 patients (27.9%) had a homozygous genotype.

Aorta and carotid IMT were similar between the two groups (*p* > 0.05) (Table 3). However, carotid strain index measurements were significantly higher in the patient group than in the control group (*p* ≤ 0.001).

Patients were divided into two groups according to the number of attacks and re-evaluated. When patients with less than three attacks and patients with three or more attacks were evaluated, no significant difference was observed between the groups (Table 4).

In addition, ESR, CRP, SAA, and fibrinogen levels among acute-phase reactants were significantly higher in the patient group than in the control group (<0.001) (Table 3). According to correlation analysis, in the patient group, the ESR was found to be positively and weakly correlated with carotid strain index measurements (*p* < 0.05). There was no significant correlation between ESR, CRP, disease duration, and number of attacks, and aortic IMT and carotid IMT measurements (Table 5).

Furthermore, the relationship between the number of attacks, disease duration, and acute-phase reactants was evaluated in the patient group. A positive correlation was observed between CRP level and both the number of attacks and disease duration. However, no significant correlation was found between the other acute-phase reactants (ESR, SAA, Fibrinogen) and the number of attacks and disease duration (Table 6).

## 4. Discussion

The objective of this study was to investigate the impact of chronic subclinical inflammation on vascular structures in patients with FMF. The results demonstrated that carotid strain index measurements were significantly higher in the patient group than in the control group. However, no significant differences were found between the groups in terms of carotid or aortic IMT. Furthermore, even during remission, ESR and CRP levels were significantly higher in the patient group than in the control group.

Multiple organ systems can be adversely affected by the activation of the inflammatory system. The vascular system is also expected to be impacted by inflammation, and studies have demonstrated a positive correlation between arterial stiffness and inflammatory markers such as CRP, IL-6, IL-1β, monocyte chemoattractant protein-1, and tumor necrosis factor-α [16,17,18]. Additionally, autoimmune rheumatic diseases such as juvenile rheumatoid arthritis, spondyloarthropathies, vasculitic syndromes, and systemic lupus erythematosus are known to be associated with early-onset arterial stiffness. This connection contributes to increased cardiovascular disease risk in these patients.

In addition to the established factors of inflammation and immunological abnormalities, other potential contributors to early vascular damage include lipoprotein levels, high blood pressure, predisposition to diabetes, being over/under-weight, presence of platelets carrying complement protein C4d, changes in endothelial cell functions, epigenetic effects, renal diseases, hyperuricemia, thyroid disorders, and vitamin deficiencies [19,20]. Vascular inflammation, both systemic and localized, is a pivotal factor in the early loss of vascular elasticity observed in autoimmune diseases. The occurrence of inflammation in the subintimal vascular and perivascular layers is more prevalent among patients diagnosed with autoimmune rheumatic diseases when compared to the general population. Vascular inflammation can contribute to arterial stiffness as a result of several factors, including edema, infiltration by inflammatory cells, fibrosis, alterations to the extracellular matrix, and modifications in the functionality of endothelial and smooth muscle cells. Increased levels of circulating complement are also associated with vascular stiffness [21,22].

In this study, carotid strain index measurements, which are used to assess arterial stiffness, were observed to be higher in the patient group than in the control group. It is hypothesized that this was associated with the persistence of subclinical inflammation during the remission phase in patients included in the study. The fact that ESR and CRP measurements were significantly higher in the patient group compared to the control group supports this hypothesis. However, proatherogenic effects occur through the induction of systemic inflammation, dyslipidemia, insulin resistance, hypercoagulability, impaired endothelial function, and oxidative stress. One of the important indicators of this damage is CRP. CRP and other biomarkers of systemic inflammation are also known to be involved in a cause–effect relationship in the process of cardiovascular disease [23]

Arterial stiffness is an early structural and functional alteration associated with atherosclerosis [24]. The findings of this study suggested that vascular changes might begin early in FMF patients, even in the absence of clinical cardiovascular pathology. The early identification of arterial stiffness may indicate the potential risk for future atherosclerotic changes. Moreover, studies have reported that as inflammation severity increases, arterial stiffness also increases [25]. In this respect, it can be expected that the longer the time spent with the disease, the higher the risk of cardiovascular disease. The potential risk in childhood remains uncertain. However, it can be postulated that the probability of premature cardiovascular incidents in adulthood may be heightened in these patients, even in the absence of frequent attacks. In a study demonstrating an increase in arterial stiffness during the attack period in FMF, it was reported that this increase was absent in patients in remission and in the control group [26]. Vampertzi et al. also evaluated arterial stiffness parameters in patients in remission and found no significant difference compared to the control group [27]. However, the elevated levels of other inflammatory markers observed in these patients thought that the inflammatory process persists during remission, which may contribute to arterial changes. Additionally, Sgouropoulou et al. found that arterial stiffness in FMF patients in remission was similar to that in the control group. They stated that this result was an indicator of a good response to colchicine treatment [28].

Additionally, endothelial dysfunction is believed to be a contributing factor in the initiation of atherosclerosis [29]. Endothelial dysfunction triggers events that increase atherosclerosis, such as vasoconstriction, leukocyte adhesion, platelet aggregation, oxidative stress, smooth muscle proliferation, and migration. Chronic inflammation activates endothelial cells, leading to the production of reactive oxygen species and the formation of foam cells and atherosclerotic plaques. Increased inflammation in FMF is associated with vasoconstriction-vasodilatation, smooth muscle cell proliferation, and imbalance in fibrinolysis, leading to endothelial dysfunction [10,30].

Although the association of increased carotid intima–media thickness (CIMT), which is an indicator of atherosclerosis in childhood, with hypertension, obesity, diabetes, and metabolic syndrome is known, the association of FMF with atherosclerosis is not fully understood. Mahmoud et al. reported that inflammation has an important role in atherogenesis. They showed that CIMT was higher in children with FMF compared to the control group [12]. Furthermore, Bilginer et al. observed that CIMT was elevated in pediatric patients with FMF. The authors attributed this increase in CIMT to increased inflammation during the remission period. In support of this, they showed that the serum levels of inflammation markers ESR, CRP, and SAA were high in the remission period in the patient group [13].

In this study, the lack of significant differences in carotid IMT and aortic IMT between the patient and control groups could be attributed to the relatively short disease duration in the patient cohort. Studies suggested that with longer disease exposure, vascular changes might become more evident [28,31]. A further reason for the absence of a notable discrepancy between the carotid and aortic IMT measurements of the patient and control groups was the markedly lower prevalence of atherosclerosis in the pediatric age group compared to adults [32]. Therefore, both the short duration of the disease and the lack of time to develop vascular complications may not have led to a significant increase in CIMT. Conversely, it has been postulated that mutated pyrin plays a pivotal role in the pathogenesis of FMF, potentially resulting in uncontrolled inflammation [33]. Pyrin or other proteins in the pathogenetic pathways of FMF may slow the development of atherosclerosis by interacting factors involved in the pathogenesis of atherosclerosis. The ineffectiveness of non-steroidal anti-inflammatory drugs and corticosteroids in treating acute episodes of FMF, coupled with the marked efficacy of colchicine in both preventing FMF attacks and halting the progression of amyloidosis, points to the possibility that the underlying inflammatory mechanisms may differ between FMF and other rheumatic disorders. Another possibility may be that the inflammation is less severe during the remission period. The absence of intense inflammation between attacks, the fact that the attacks are not severe due to the effect of colchicine, and remission within an average of 3 days may prevent the development of atherosclerosis, especially in childhood [34]. Additionally, the use of colchicine, which has anti-inflammatory effects, may have played a role in suppressing arterial damage [35]. The fact that this drug improves some symptoms, such as fatigue, decreased appetite and/or activity, and sleep disturbance, which may be due to ongoing subclinical inflammation during remission, may also be interpreted as reducing vascular damage that may develop [36].

This study has several limitations. Firstly, renal biopsy was not performed in any of the patients, as none of them were considered to have amyloidosis due to the absence of clinical and laboratory proteinuria. When patients were grouped according to the number of attacks, no difference may have been found between the groups because CRP and SAA are general markers of inflammation. However, if biomarkers with acute up-regulation properties, such as TNFα and IL-1β, were examined, this difference could have been shown. Another limitation was that the study was designed as a single-center study with a limited number of patients. The final limitation was that the elastographic evaluation was performed by a single operator. Future studies with multiple independent readers and larger, multicenter cohorts are needed to confirm these results.

This study demonstrated that chronic subclinical inflammation in pediatric FMF patients has a significant effect on arterial stiffness. Even during remission, ongoing inflammation in children with FMF appears to contribute to vascular alterations, particularly increased arterial stiffness. The early identification of these vascular changes is crucial for predicting long-term cardiovascular risks, including the development of atherosclerosis. Regular arterial ultrasonographic and elastographic assessments may serve as noninvasive and effective methods for monitoring endothelial damage and preventing future cardiovascular complications in pediatric FMF patients. These findings support the need for early cardiovascular monitoring and intervention in children with FMF.

## Figures and Tables

**Table 1:** Demographic characteristics of the patient and control groups.

Characteristics	Patient Group (*n* = 43) (IQR)	Control Group (*n* = 50) (IQR)	*p*-Value
Age, y	9.1 (6; 14)	10.8 (7; 13)	0.129
Sex (M/F) (*n*, %)	21 (48.8)/22 (51.2)	21 (42.0)/29 (58)	0.511
Weight, kg (range)	31.10 (20.00; 48.00)	36.20 (23.15; 53.30)	0.252
Height, cm (range)	134.40 (120.30; 155.60)	139.40 (125.26; 159.60)	0.311

Sex is summarized by number (*n*) and frequency (%). The median [IQR (25–75)] are given. y: years, M: Male, F: Female.

**Table 2:** Clinical characteristics of the patients.

Characteristics	Median (IQR)	Min-Max
Disease duration, years	3.1 (2; 6)	1–10
Number of attacks	6 (2; 12)	1–12

**Table 3:** Ultrasonography and laboratory test results of the patient and control groups.

Measurements	Patient Group (*n* = 43)	Control Group (*n* = 50)	*p*-Value
Aorta intima–media thickness (mm)	0.05 (0.04; 0.05)	0.05 (0.04; 0.05)	0.203
Carotid intima–media thickness (mm)	0.04 (0.04; 0.05)	0.04 (0.04; 0.05)	0.152
Carotid SI	0.5 (0.4; 0.6)	0.4 (0.4; 0.5)	0.001
ESR (mm/h)	22.0 (12.0; 33.0)	6.0 (3.0; 9.0)	<0.001
CRP (mg/dL)	0.80 (0.27; 4.25)	0.05 (0.05; 0.09)	<0.001
SAA (μg/mL)	0.15 (0.08; 0.64)	0.07 (0.02; 0.32)	<0.001
Fibrinogen (mg/dL)	303 (258; 371)	217 (203; 247)	<0.001
Urea (mg/dL)	22.4 (10.3; 30.9)	19.8 (12.6; 28.1)	0.304
Creatinine (mg/dL)	0.5 (0.3; 0.6)	0.4 (0.3; 0.5)	0.218
AST (IU/L)	14 (11; 28)	16 (12; 32)	0.154
ALT (U/mL)	11 (9; 16)	10 (8; 14)	0.329
Triglyceride (mg/dL)	84 (70; 96)	78 (65; 83)	0.267
Total cholesterol (mg/dL)	148 (115; 173)	137 (108; 171)	0.348

The medians (IQRs) were given. SI: strain index, ESR: erythrocyte sedimentation rate, CRP: C-reactive protein. SAA: serum amyloid A. AST: aspartate aminotransferase. ALT: alanine aminotransferase.

**Table 4:** Ultrasonography and laboratory test results of the patient according to the attacks number.

Measurements	Attacks Number < 3 (*n* = 17)	Attacks Number ≥ 3 (*n* = 26)	*p*-Value
Aorta intima–media thickness (mm)	0.05 ± 0.009	0.04 ± 0.004	0.078
Carotid intima–media thickness (mm)	0.04 ± 0.005	0.04 ± 0.007	0.739
Carotid SI	0.56 ± 0.22	0.57 ± 0.21	0.919
ESR (mm/h)	19.76 ± 9.51	25.10 ± 14.53	0.185
CRP (mg/dL)	1.73 ± 3.28	3.21 ± 3.48	0.165
SAA (μg/mL)	0.96 ± 2.16	0.86 ± 1.90	0.872
Fibrinogen (mg/dL)	312.47 ± 70.91	306.96 ± 88.94	0.830
Urea (mg/dL)	30.04 ± 6.82	28.92 ± 5.83	0.562
Creatinine (mg/dL)	0.55 ± 0.13	0.52 ± 0.11	0.411
AST (IU/L)	36.11 ± 8.01	37.25 ± 9.02	0.669
ALT (U/mL)	26.76 ± 10.98	23.62 ± 8.81	0.345
Triglyceride (mg/dL)	76.74 ± 12.32	78.71 ± 18.19	0.174
Total cholesterol (mg/dL)	137.68 ± 28.94	128.58 ± 38.78	0.347

Mean ± SD were given. SI: strain index, ESR: erythrocyte sedimentation rate, CRP: C-reactive protein. SAA: serum amyloid A. AST: aspartate aminotransferase. ALT: alanine aminotransferase.

**Table 5:** Correlations between aortic intima–media thickness, carotid intima–media thickness, and carotid strain index measurements in the patient group.

Test Results and Clinical Characteristics	Aorta IMT	Carotid IMT	Carotid SI
ESR (mm/h)	−0.032	−0.190	**0.364**
CRP (mg/dL)	−0.155	−0.301	0.045
Disease duration, years	−0.134	−0.022	0.256
Number of attacks	−0.262	−0.061	0.058

The bold coefficient was significant at the 0.05 level. All values were Spearman’s rho correlation coefficients. ESR: erythrocyte sedimentation rate, CRP: C-reactive protein, IMT: intima–media thickness, SI: strain index.

**Table 6:** Correlation between acute-phase reactants, disease duration, and the number of attacks in the patient group.

Measurements	Number of Attacks	Disease Duration, Years
CRP (mg/dL)	0.33 *	0.44 **
ESR (mm/h)	0.11	0.29
SAA (μg/mL)	−0.58	0.07
Fibrinogen (mg/dL)	−0.047	0.09

* Correlation is significant at the 0.05 level. ** Correlation is significant at the 0.01 level. All values were Spearman’s rho correlation coefficients.

## Data Availability

The data that support the findings of this study are available from the corresponding author due to ethical reasons.
